# Correlative Factors of the Deterioration of Necrotizing Enterocolitis in Small for Gestational Age Newborns

**DOI:** 10.1038/s41598-017-18467-8

**Published:** 2018-01-08

**Authors:** Lijuan Luo, Wenbin Dong, Lingping Zhang, Xuesong Zhai, Qingping Li, Xiaoping Lei

**Affiliations:** Department of Newborn Medicine, the Affiliated Hospital of Southwest Medical University, Luzhou, Sichuan China

## Abstract

Small for gestational age (SGA) infants have an increased risk of necrotizing enterocolitis (NEC), but SGA has been found to not be a risk factor for the deterioration of NEC in previous literature. Few studies have focused on correlative factors of the progression of NEC in SGA newborns. The present retrospective observational study was performed in 64 SGA infants with Bell’s stage II NEC. The dependent variable was Bell’s stage II NEC that progressed to stage III after diagnosis. A stepwise forward multivariate logistic regression model was used to select potential correlative factors for the progression of NEC in SGA newborns. The results showed that elevation of CRP after NEC diagnosis (aOR 39.21, 95% CI 6.62–249.2) has an increased risk for deteriorating Bell’s stage II NEC. In contrast, NEC in infants with congenital heart disease had a decreased risk of deterioration (aOR 0.11, 95% CI 0.01–0.92). Our findings indicated that serial CRP measurements post NEC diagnosis may be useful in predicting the deterioration of NEC.

## Introduction

Necrotizing enterocolitis (NEC) is one of the most frequent life-threatening gastrointestinal inflammatory diseases in newborn infants^1^. It occurs in approximately 1–8% of newborns in the neonatal intensive care unit (NICU), with an estimated mortality rate that ranges from 20 to 30%^2^. It has been reported that surgical therapy was needed in 27 to 52% of newborns with NEC^3^, and the post-operative mortality was more than 40%^4^. A newborn with progressive NEC will be more likely to undergo surgical intervention, subsequently leading to higher mortality, higher hospital cost, and higher risks of long-term adverse outcomes^5^.

It was well established that shorter gestational age and lower birth weight were the most common risk factors for the deterioration of NEC^5,6^. Small for gestational age (SGA), birth weight less than the 10^th^ percentile of specific-gestational age, which combines both birth weight and gestational age, had been reported to be associated with increased mortality and morbidity, including NEC^7,8^. However, SGA has been found to not be a risk factor for the deterioration of NEC in previous literatures^5^. To the best of our knowledge, few studies focused on the correlative factors of the progression of NEC in SGA newborns. The present study aimed to explore the potential influencing factors that are associated with the progression of NEC in SGA newborns.

## Methods

### Study Population

The Affiliated Hospital of Southwest Medical University is one of the tertiary referral centers in southwest China. The NICU received almost all of the high-risk newborns in the neighborhood area of the hospital. In the present study, the eligible study subjects were SGA newborns with a confirmed diagnosis of Bell’s stage II NEC^9^ between Jan 2010 to Mar 2017. SGA was defined as patients with a birth weight less than the 10^th^ percentile for gestational age according to the growth curves for Chinese newborns by separate standards for singletons^10^ and twin fetuses^11^. SGA was classified into two groups based on the percentile of the birth weight for specific gestational age: ≤ 3rd percentile and >3^rd^, <10th percentile^10,11^.

**Figure 1:**
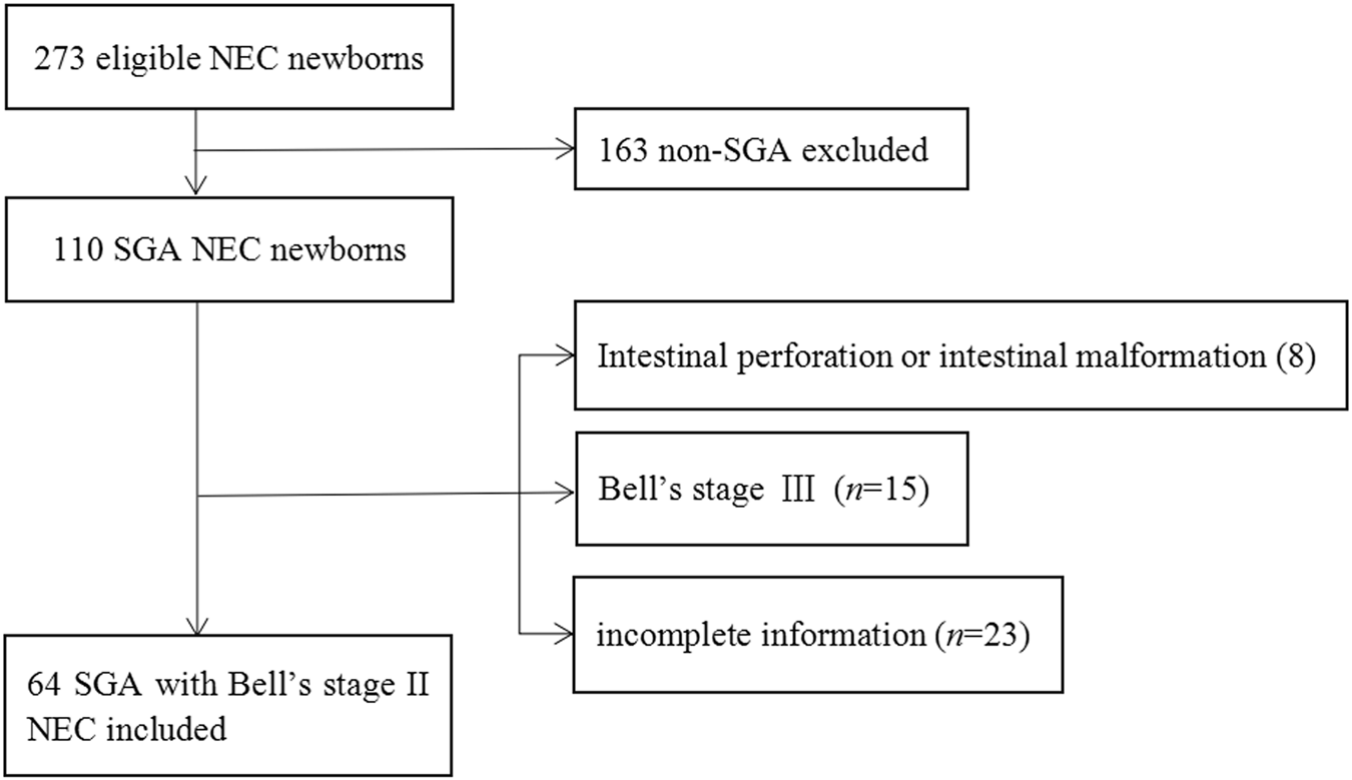
Flow Chart in the Selection of SGA infants with Bell’s stage II NEC.

Of the 273 newborns with confirmed diagnosis of NEC in the study period, 163 non-SGA newborns were excluded. At the diagnosis of NEC, infants with spontaneous intestinal perforation or intestinal malformation (aproctia, intestinal atresia, Hirschsprung’s disease) (*n* = 8), with Bell’s stage III at diagnosis (*n* = 15) or with incomplete information (*n* = 23) were also excluded. In total, 64 SGA infants with Bell’s stage II NEC met the inclusion criterion (as seen in Fig. 1).

### Diagnosis and treatment of NEC

NEC subjects were staged according to the criteria proposed by Bell and modified by Walsh^9^. Stage II NEC was defined according to two criteria: onset of at least one of the below clinical symptoms: abdominal distension, emesis, and occult or gross blood in the stool (without fissure); and radiographic or ultrasound findings of pneumatosis intestinalis or portal vein gas. Stage III NEC was defined according to the above clinical symptoms plus radiographic or ultrasound findings of pneumoperitoneum or large amounts of ascites or someone who requires bowel surgery if medical therapy was not valid within 48 hours^5,9^. All NEC newborns underwent the same basic treatment protocol including bowel rest, intravenous nutrition, parenteral antibiotics, nasogastric suction and intensive care therapy (cardio-respiratory support and blood product transfusion) if necessary.

### Data Collection

The present research was a retrospective observational study. Data were extracted from the hospital’s patient database; all collected data was anonymized. Informed consent and ethical approval is not needed for using the anonymized clinical data. Once the NEC case was identified and included in the present study, all physician and nurse notes pertaining to NEC, laboratory examinations, radiographic and ultrasonic reports, and surgical records were reviewed.

The following maternal and neonatal baseline characteristics were collected: maternal pregnancy-induced hypertension (PIH) (yes/no), maternal diabetes (yes/no), vaginal delivery (yes/no), prolonged rupture of membrane (PROM) > 18 h (yes/no), amniotic fluid contamination (yes/no), perinatal asphyxia (yes/no), gestational age at birth, birth weight, gender (male/female), multiple gestations (yes/no), mode of feeding at home (exclusively breast, exclusively bottle or mixed feeding), age of NEC onset (the day in which at least one of the following signs or symptoms appeared: pre-feeding gastric residuals, emesis, abdominal distension, or bloody stool), age of NEC diagnosis (the day of the radiographic or ultrasound findings meet the diagnostic criteria of Bell’s stage II), and complications of NEC newborns (metabolic acidosis, intracranial hemorrhage, sclerema neonatorum, abnormal glycemia, sepsis, etc.). In the present study, tiny-to-small PDA or patent foramen ovale were not included in congenital heart disease (CHD) because of the ubiquity of small ductuses in the neonatal population and the lack of evidence of decreased splanchnic perfusion from the ductal steal^12^.

The reviewed laboratory test included: all white blood cell count, platelet count, C-reactive protein (CRP) and all of the radiographic and ultrasonic reports during hospitalization. The white blood cell count, platelet count and CRP that were tested within 24 hours of NEC diagnosis were considered to be the baseline value. CRP levels were measured in all infants in the hours following the diagnosis of NEC, and this test was repeated at least once every 24 h for the first 48 h, every 3–5 days for the following days, and additional measurements were conducted based on infant condition. If the highest serum CRP level from diagnosis of NEC until hospital discharge was higher than the baseline CRP value, it was defined as elevated CRP post NEC diagnosis.

The recorded treatment protocols included: days for first cessation of enteral feeding, days for first nasogastric suction, days for antibiotics (broad spectrum antibiotics: β-lactams, anaerobic antimicrobial therapy: metronidazole), and probiotics: bifidobacterium, dopamine support, transfusion: red blood cell, blood plasma, cryoprecipitate, etc.

### Statistical Analysis

The normality of the continuous variables was tested before the data analysis. Normally distributed variables were described as the mean ± standard deviation (*mean* ± *SD*) and analyzed using an independent 2-tailed *t*-test. Skewed variables were described as median (interquartile range, *IQR*) and analyzed by *Mann-Whitney U* tests. Categorical variables were analyzed by chi-square tests or Fisher’s exact test. A stepwise forward multivariate logistic regression model was used to select the potential correlative factors of the progression of NEC. The variables with statistical significance (*P* value < 0.05) in the univariate model were used to calculate the adjusted odds ratios (aOR) and the corresponding 95% confidential intervals (CI) in a stepwise forward model of multivariate logistic regression. In clinical practice, more CRP measurements were done in the sicker infants and may raise the possibility of ascertainment bias in this retrospective study. To avoid this bias, we further reanalyzed the data in those NEC cases without missing the CRP measurements on the 1st, 2nd, and 7th day post diagnosis. The variables were processed with SPSS13.0 (SPSS Inc., Chicago, IL) using descriptive and inferential statistics.

## Results

A total of 64 SGA newborns with Bell’s stage II NEC met the inclusion criteria; more than 60% (40/64) of them were full-term infants. Overall, 15 of the 64 infants (25%) deteriorated to Bell’s stage III during hospitalization, and more than 50% (8/15) of them received surgical treatment. Approximately 70% (44/64) of the neonates were transferred from other hospitals. A total of 42 infants received anaerobic antimicrobial therapy in this study.

The demographic features of SGA newborns with Bell’s stage II NEC that progressed to Bell’s Stage III or not are summarized in Table 1. Lower birth weight (1920 g ± 450 g *vs*. 2284 g ± 420 g, *P* = 0.005) and shorter gestational age (36.0 w ± 1.8 w *vs*.37.8 w ± 1.9 w, *P* = 0.002) existed in newborns with NEC that progressed to Bell’s stage III than those without. There were no significant differences in gender, age of onset and diagnosis of NEC, mode of delivery, mode of feeding at home, SGA severity (≤3rd percentile and >3^rd^, <10^th^ percentile^10,11^), and those transferred into the center or born in the center between the two groups.

**Table 1:** Demographic Features of Small for Gestational Age Neonates with Necrotizing Enterocolitis Who Progressed to Stage III or Not.

	Stage III (n = 15)	No stage III (n = 49)	*P*
Pregnancy induced hypertension (n, %)	4 (26.7)	9 (18.4)	0.48
Antenatal use of corticosteroids (n, %)	1 (6.7)	0	0.23
Amniotic fluid contamination (n, %)	1 (6.7)	7 (14.3)	0.67
Rupture of membranes >18 hours (n, %)	1 (6.7)	1 (2.0)	0.42
Vaginal delivery (n, %)	6 (40.0)	14 (28.6)	0.53
Breast fed at home (n, %)	2 (13.3)	10 (20.4)	0.60
Male (n, %)	9 (60.0)	25 (51)	0.54
Multiple gestations (n, %)	5 (33.3)	7 (14.3)	0.20
SGA severity: ≤ 3rd percentile (n, %)	10 (66.7)	23 (46.9)	0.18
Transferred in infants (n, %)	9 (60.0)	35 (79.5)	0.61
Onset age [days, median (IQR)]	5 (2–7)	5 (1–10)	0.67
Diagnosis age (days, mean ± SD)	9.8 ± 6.2	10.3 ± 7.3	0.79
Birth weight (grams, mean ± SD)	1920 ± 450	2284 ± 420	0.005
Gestational age (weeks mean ± SD)	36.0 ± 1.8	37.8 ± 1.9	0.002

SD: standard deviation; IQR: interquartile range.

Table 2 showed the incidence difference of neonatal complications between the two groups. There are 23 infants with CHDs in the present study: isolated atrial septal defect (ASD, *n* = 13), isolated patent ductus arteriosus (PDA, *n* = 6), ventricular septal defect (VSD) in addition to ASD (*n* = 1), VSD in addition to PDA (*n* = 1), and three types of cardiac lesions (ASD, VSD and PDA, *n* = 2). Less frequency of CHD occurred in newborns with NEC that progressed to Bell’s stage III than those without (13.3% *vs*. 42.9%, *P* = 0.04).

**Table 2:** Complications of Small for Gestational Age Neonates with Necrotizing Enterocolitis Who Progressed to Stage III or Not.

	Stage III(*n* = 15)	No stage III (*n* = 49)	*P*
Congenital heart disease* (n, %)	2 (13.3)	21 (42.9)	0.04
Pathoglycemia (n, %)	2 (13.3)	3 (6.1)	0.72
Sepsis (n, %)	7 (46.7)	12 (24.5)	0.19
Coagulopathy (n, %)	5 (33.3)	22 (44.9)	0.43
Sclerema neonatorum (n, %)	3 (20.0)	7 (14.3)	0.90
Intracranial hemorrhage (n, %)	1 (6.7)	8 (16.3)	0.61
Metabolic acidosis (n, %)	2 (13.3)	4 (8.2)	0.92
Apnea (n, %)	0	1 (2.0)	1
Hemolytic disease of newborn (n, %)	1 (6.7)	9 (18.4)	0.49
Asphyxia (n, %)	0	2 (4.1)	1
Anemia (n, %)	5 (33.3)	21 (42.9)	0.51
Liver dysfunction (n, %)	0	4 (8.2)	0.57
Renal dysfunction (n, %)	2 (13.3)	6 (12.2)	1.0

*Tiny-to-small PDA or patent foramen ovale were not defied as congenital heart disease.

Table 3 showed the comparisons of the treatment protocol and laboratory tests of the two groups. We found that a longer time for nasogastric suction (6 d *vs*. 2 d, *P* = 0.04), abnormal WBC count (<5 × 10^9^/L or >20 × 10^9^/L, 40% *vs*. 10.2%, *P* = 0.02) and elevated CRP post NEC diagnosis (80% *vs*. 14.3%, *P* = 0.0001) occurred more often in newborns with NEC that progressed to Bell’s stage III than those without. There were no significant differences in blood product transfusion, anaerobic antimicrobial therapy, time of broad spectrum antibiotics, and abnormal CRP value at diagnosis in the two groups. Since surgical intervention may influence the concentration of serum CRP, we excluded the newborns who received surgical treatment and found that the incidence of elevated CRP post NEC diagnosis was still significantly higher in newborns with NEC that progressed to Bell’s stage III than those without (71.4% *vs*. 14.3%, *P* = 0.003).

**Table 3:** Treatment Protocol and Laboratory Test of Small for Gestational Age Neonates with Necrotizing Enterocolitis Who Progressed to Stage III or Not.

	Stage III (*n* = 15)	No stage III (*n* = 49)	*P*
Albumin (n, %)	8 (53.3)	12 (24.5)	0.07
Blood transfusion (n, %)	9 (60.0)	18 (36.7)	0.11
Intravenous immunoglobulin (n, %)	2 (13.3)	5 (10.2)	0.32
Probiotics (n, %)	4 (26.7)	23 (46.9)	0.16
Anaerobic antimicrobial therapy (n, %)	11 (73.3)	31 (63.3)	0.47
Dopamine support (n, %)	5 (33.3)	13 (26.5)	0.95
Broad spectrum antibiotics use (days, mean ± SD)	11.9 ± 5.5	13.5 ± 6.9	0.42
Cessation of enteral feeding (days, mean ± SD)	7.0 ± 3.3	7.2 ± 4.8	0.89
Nasogastric suction [days, median(IQR)]	6 (1–8)	2 (0–5)	0.04
WBC count* <5 × 10^9^/L or >20 × 10^9^/L (n, %)	6 (40)	5 (10.2)	0.02
Platelet count* <100 × 10^9^/L (n, %)	3 (20)	5 (10.2)	0.58
CRP* >8 mg/L (n, %)	4 (26.7)	12 (24.5)	1
Elevated CRP post NEC diagnosis, %(n)	12 (80.0)	7 (14.3)	0.0001

*Baseline values were tested within 24 hours of NEC diagnosis; WBC: white blood cell; CRP: C-reactive protein; NEC: necrotizing enterocolitis; SD: standard deviation; IQR: interquartile range.

In the multivariate logistic regression model, low birth weight (aOR 10.76, 95% CI 0.82–141.78) and elevated CRP after NEC diagnosis (aOR 39.21, 95% CI 6.62–249.2) seem to have increased risks for NEC deterioration, while NEC infants with CHD had a decreased risk of deterioration (aOR 0.11, 95% CI 0.01–0.92) (Table 4).

**Table 4:** Correlative Factors for Necrotizing Enterocolitis Deterioration in SGA Newborns.

		Stage III (n = 15)		No stage III (n = 49)		OR (95% CI)	*P*	aOR (95% CI)	*P*
Preterm (<37 w)(n, %)		10 (66.7%)		14 (28.6%)		5.00 (1.45–17.27)	<0.01	—	—
Birth weight (<2500 g)(n, %)		14 (93.3%)		33 (67.3%)		6.79 (0.82–56.26)	<0.05	10.76 (0.82–141.78)	0.07
Congenital heart disease (n, %)		2 (13.3%)		21(42.9%)		0.20 (0.04–1.01)	<0.05	0.11 (0.01–0.92)	<0.05
WBC count <5 × 10^9^/L or >20 × 10^9^/L (n, %)		6 (40%)		5 (10.2%)		5.87 (1.47–23.47)	<0.05	—	—
Elevation of CRP after NEC diagnosis (n, %)		12 (80%)		7 (14.3%)		24.00 (5.37–107.23)	<0.001	39.21 (6.62–249.20)	<0.001
Time for nasogastric suction[days, median (IQR)]		6 (1–8)		2 (0–5)		—	<0.05	—	—

WBC: white blood cell; CRP: C-reactive protein; NEC: necrotizing enterocolitis; IQR: interquartile range; OR: odds ratio; CI: confidence interval; a OR: adjusted odds ratio.

While reanalyzing the data in those NEC cases without missing the CRP measurements on the 1st, 2nd, and 7th day post diagnosis, a similar association exists between the elevation of CRP after NEC diagnosis and NEC deterioration (aOR 11.7, 95% CI 1.1–122.4) (see supplement table).

## Discussion

In the present study, the findings seem to indicate that low birth weight and elevated CRP after NEC diagnosis are correlative factors for NEC progressing from stage II to stage III in SGA infants, and NEC infants with CHD have a decreased risk of NEC deterioration.

CRP is an acute phase reactant that is produced in response to inflammation caused by infection or tissue injury. During the acute phase response, CRP increases rapidly within several hours and reaches a peak at approximately 48 hours. With a short half-life, once the stimulant resolves, the concentration of serum CRP will rapidly decline to a normal level^13^. In a previous study^14^ that focused on the predictive value of serial CRP measurements in newborns with suspected NEC, it has been reported that elevated CRP indicates definite NEC, and persistently elevated CRP after initiation of appropriate medical management suggests the progression of NEC. In our study, there was no difference in serum CRP concentrations at NEC diagnosis, while the incidence of elevated CRP post NEC diagnosis occurred at a significantly higher rate in infants who progressed to Bell’s stage III than those without. In *Srinivasjois R*’s study, in which all cases of NEC required surgery, CRP remained elevated even after initiation of appropriate medical therapy^15^. In this study, in the surgical newborns, nearly 90% (7/8) of NEC cases had an elevated CRP after NEC diagnosis. Since surgical intervention can influence the concentration of serum CRP^16^, we further compared NEC cases that progressed to Bell’s stage III with NEC cases that did not progress to stage III in infants who only received medical treatment and found that elevated CRP post NEC diagnosis was still significantly associated with the progression of NEC. In clinical practice, antibiotics are strongly recommended for NEC management. Theoretically, the CRP value could be decreased by using antibiotics to treat the bacterial infection. However, *Autmizguine* ^17^ and our previous study^18^ found that broad-spectrum antibiotics plus anaerobic antimicrobial therapy cannot prevent NEC newborns from deteriorating. In this study, we also found that the duration for broad-spectrum antibiotics with anaerobic antimicrobial agents have no significant influence on the progression of NEC in SGA newborns. Since more CRP measurements were done in the sicker infants in clinical practice, this may increase the possibility of ascertainment bias in this retrospective study. However, while reanalyzing the data in those NEC cases without missing the CRP measurements on the 1st, 2nd, and 7th day post diagnosis, a similar association between the elevated CRP after NEC diagnosis and NEC deterioration also existed.

Low birth weight is the most common risk factor for the development and prognosis of NEC. Two large cohort studies reported that newborns with the lowest birth weight have the highest incidence of NEC^6,19^. Another study found that, when NEC is onset, newborns with lower birth weights are more likely to become severe and need surgery^5^. Our findings in the present study are consistent with these studies. Although there was no statistical significance in our study, it could be explained by the small number of NEC cases; the large OR value (10.76) indicated its clinical significance.

In newborns with CHD, the pathophysiological mechanism of NEC may be different from that in preterm babies and is proved to be associated with the initiation of enteral feeding in the context of functionally immature gut mucosa, bowel injury, and pathogenic organisms^20^. In CHD-related NEC, decreased mesenteric perfusion resulting from cardiovascular abnormalities may be the predominant initiating factor rather than feed initiation or bowel immaturity^12^. Animal models of NEC in asphyxiated newborn piglets demonstrated mesenteric flow insufficiency rather than gut immaturity as the predisposing factor for NEC development^21,22^. A CHD and non-CHD NEC cohort study showed that both preterm and term infants with CHD had decreased NEC-related morbidity and mortality compared with infants without CHD; the CHD group had a significantly decreased risk of perforation, need for a bowel operation, and need for a stoma^12^. Our findings are consistent with these previous studies. Different pathophysiological mechanisms between CHD-NEC and non-CHD NEC may be the potential explanation for better outcomes in CHD newborns with NEC. No infants with complex CHD are included in the present study; this might be the result of low morbidity, termination of treatment or death before developing NEC.

There are some limitations in our study, such as the inherent errors and bias of retrospective studies. Most suspected NEC cases (Bell’s stage I NEC^9^) followed a benign and uncomplicated course, and we excluded all suspected NEC cases, resulting in a small number of SGA-NEC cases included in this study. Because of the small number of NEC cases, we did not further classify the included cases to Bell’s stage IIa, IIb, IIIa, and IIIb. A previous study reported that a lack of early colostrum feeding would contribute to the deterioration of NEC^5^. The formula was used for all infants during hospitalization; we do not know what extent of formula feeding influenced our results.

In conclusion, low birth weight and elevated CRP post NEC diagnosis are associated with the deterioration of NEC, while the anaerobic antimicrobial therapy seems to have no effect on preventing the deterioration of NEC in SGA newborns; progression of NEC is less common in infants with CHD. These findings may still shed some light on clinical practice. Once Bell’s stage II NEC was diagnosed, serial measurement of serum CRP should be obtained.

## Electronic supplementary material

Supplementary Table
