# Supplementary material for: Correlative Factors of the Deterioration of Necrotizing Enterocolitis in Small for Gestational Age Newborns
Source: Sci Rep. 2018 Jan 8;8:13. doi: 10.1038/s41598-017-18467-8 (PMC5758570; doi:10.1038/s41598-017-18467-8)
Supplement: Supplementary file 1 — Supplementary Table [file 41598_2017_18467_MOESM1_ESM.pdf]

Correlative Factors of the Deterioration of Necrotizing Enterocolitis in Small for Gestational Age Newborns

Lijuan Luo, Wenbin Dong, Lingping Zhang, Xuesong Zhai, Qingping Li, Xiaoping Lei*

Supplement Table. Correlative Factors for Necrotizing Enterocolitis Deterioration in SGA Newborns without Missing CRP Measurements on the 1st, 2nd, and 7th Days post Diagnosis.

	Stage III (n = 6)	No stage III (n = 20)	*P*	aOR (95% CI)	*P*
Blood transfusion (n, %)	5 (83.3)	6 (30.0)	0.05	-	-
Elevation of CRP after NEC diagnosis (n, %)	5 (83.3)	6 (30.0)	0.02	11.7 (1.1-122.4)	0.04

CRP: C-reactive protein; NEC: necrotizing enterocolitis; OR: odds ratio; CI: confidence interval; aOR: adjusted odds ratio.
